# Reduction of circulating FABP4 level by treatment with omega-3 fatty acid ethyl esters

**DOI:** 10.1186/s12944-016-0177-8

**Published:** 2016-01-12

**Authors:** Masato Furuhashi, Shinya Hiramitsu, Tomohiro Mita, Akina Omori, Takahiro Fuseya, Shutaro Ishimura, Yuki Watanabe, Kyoko Hoshina, Megumi Matsumoto, Marenao Tanaka, Norihito Moniwa, Hideaki Yoshida, Junnichi Ishii, Tetsuji Miura

**Affiliations:** Department of Cardiovascular, Renal and Metabolic Medicine, Sapporo Medical University School of Medicine, S-1, W-16, Chuo-ku, Sapporo, 060-8543 Japan; Hiramitsu Heart Clinic, Shiroshita-cho 2-35, Minami-ku, Nagoya, 457-0047, Aichi Japan; Department of Joint Research Laboratory of Clinical Medicine, Fujita Health University School of Medicine, Toyoake, 470-1192, Aichi Japan

**Keywords:** Adipokine, Fatty acid-binding protein 4, Adipocyte, Eicosapentaenoic acid, Docosahexaenoic acid

## Abstract

**Background:**

Fatty acid-binding protein 4 (FABP4/A-FABP/aP2) mainly expressed in adipocytes is secreted and acts as an adipokine. Increased circulating FABP4 level is associated with obesity, insulin resistance and atherosclerosis. However, little is known about the modulation of serum FABP4 level by drugs including anti-dyslipidemic agents.

**Methods:**

Patients with dyslipidemia were treated with omega-3 fatty acid ethyl esters (4 g/day; *n* = 14) containing eicosapentaenoic acid (EPA) and docosahexaenoic acid (DHA) for 4 weeks. Serum FABP4 level was measured before and after treatment. Expression and secretion of FABP4 were also examined in mouse 3T3-L1 adipocytes treated with EPA or DHA.

**Results:**

Treatment with omega-3 fatty acid ethyl esters significantly decreased triglycerides and serum FABP4 level (13.5 ± 1.5 vs. 11.5 ± 1.1 ng/ml, *P* = 0.017). Change in FABP4 level by omega-3 fatty acids was negatively correlated with change in levels of EPA + DHA (r = −0.643, *P* = 0.013), EPA (r = −0.540, *P* = 0.046) and DHA (r = −0.650, *P* = 0.011) but not change in the level of triglycerides or other fatty acid composition. Treatment of 3T3-L1 adipocytes with EPA or DHA had no effect on short-term (2 h) secretion of FABP4. However, gene expression and long-term (24 h) secretion of FABP4 were significantly reduced by treatment with EPA or DHA.

**Conclusions:**

Omega-3 fatty acids decrease circulating FABP4 level, possibly by reducing expression and consecutive secretion of FABP4 in adipocytes. Reducing FABP4 level might be involved in suppression of cardiovascular events by omega-3 fatty acids.

## Background

Fatty acid-binding proteins (FABPs) are predominantly cytosolic proteins that bind hydrophobic ligands, such as long chain fatty acids [1–3]. It has been proposed that FABPs promote the transport of fatty acids to several organelles in the cell [1]. Fatty acid-binding protein 4 (FABP4), also referred to as adipocyte FABP (A-FABP) or aP2, is expressed in adipocytes and macrophages. Previous studies using FABP4-deficient mice have demonstrated that FABP4 plays important roles in the development of insulin resistance, diabetes mellitus and atherosclerosis [4–7], and chemical inhibition of FABP4 might be a novel therapeutic agent against insulin resistance, diabetes mellitus and atherosclerosis [8].

It has recently been shown that FABP4 is secreted from adipocytes via a non-classical secretion pathway during lipolysis [9–12], though the sequence of FABP4 lacks signal peptides [1]. Furthermore, FABP4 has been demonstrated to act as an adipokine for the development of insulin resistance in liver [10], suppression of cardiomyocyte contraction [13], inhibition of endothelial nitric oxide synthase in endothelial cells [14] and proliferation and migration of vascular smooth muscle cells [15]. It has also been shown that an elevated serum level of FABP4 is associated with obesity, insulin resistance, hypertension, cardiac dysfunction, atherosclerosis and cardiovascular events [9, 16–23]. However, little is known about the alteration of FABP4 level by drugs including anti-dyslipidemic agents.

It has been reported that atorvastatin, a cholesterol-lowering statin, decreases FABP4 level [24]. Furthermore, omega-3 fatty acids have been reported to inhibit adipocyte differentiation and lipid accumulation *in vitro*, possibly leading to a decrease in the expression of FABP4, also known as a differentiation marker of adipocytes [25–27]. We hypothesized that modulation of lipid levels by drugs can decrease the circulating FABP4 level. In this study, we investigated the effect of omega-3 fatty acid ethyl esters containing eicosapentaenoic acid (EPA) and docosahexaenoic acid (DHA) on serum FABP4 level in patients with dyslipidemia. We also examined drug-induced regulation of the expression and secretion of FABP4 in adipocytes in the presence or absence of lipolytic stimulation.

## Methods

Human study (Study 1) conformed to the principles outlined in the Declaration of Helsinki and was performed with the approval of the Ethical Committee of Fujita Health University. Written informed consent was received from all of the subjects. Experimental procedures for *in vitro* study using mouse 3T3-L1 adipocytes (Study 2) were performed with approval from the Animal Care and Experiment Committee of Sapporo Medical University.

### Study 1: Effects of omega-3 fatty acids on serum FABP4 level

Male patients with dyslipidemia (*n* = 14; mean age: 40.2 ± 1.7 years) were enrolled from outpatient clinics affiliated with Fujita Health University. Entry criteria were no treatment with lipid-lowering agents, anti-diabetic agents or antihypertensive agents and absence of complications such as hepatic disease, cerebrovascular or cardiovascular disease, or renal disease. Patients were treated with 4 g (2 g twice daily; after breakfast and dinner) omega-3 fatty acid ethyl esters containing 1,860 mg/day EPA ethyl ester and 1,500 mg/day DHA ethyl ester for 4 weeks in outpatient clinics.

### Measurements

Body mass index (BMI, kg/m^2^) was calculated as body weight divided by the square of body height. Before and after the 4-week treatment with omega-3 fatty acid ethyl esters, blood samples were collected after an overnight fast, and serum and plasma samples were analyzed immediately or stored at −80 °C until biochemical analyses. Serum FABP4 concentration was measured using an enzyme-linked immunosorbent assay kit (Biovendor R&D, Modrice, Czech Republic). Precision, accuracy and reproducibility of the kit have been described previously [9]. Hemoglobin A1c (HbA1c) was determined by a latex coagulation method and was expressed in NGSP scale. Plasma glucose and fasting plasma insulin was measured by the glucose oxidase method and a radioimmunoassay method, respectively. Levels of creatinine (Cr), triglycerides, total cholesterol and high-density lipoprotein (HDL) cholesterol were determined by enzymatic methods. Level of low-density lipoprotein (LDL) cholesterol was calculated by the Friedewald equation. The compositions of 24 fatty acids, including lauric acid (C12:0), myristic acid (C14:0), myristoleic acid (C14:1ω5), palmitic acid (C16:0), palmitoleic acid (C16:1ω7), stearic acid (C18:0), oleic acid (C18:1), linoleic acid (C18:2ω6), γ-linolenic acid (C18:3ω6), α-linolenic acid (C18:3ω3), arachidic acid (C20:0), eicosenoic acid (C20:1ω9), eicosadienoic acid (C20:2ω6), eicosatetraenoic acid (C20:3ω9), dihomo-γ-linolenic acid (C20:3ω6), arachidonic acid (C20:4ω6), EPA (C20:5ω3), behenic acid (C22:0), erucic acid (C22:1ω9), docosatetraenoic acid (C22:4ω6), docosapentaenoic acid (C22:5ω3), lignoceric acid (C24:0), DHA (C22:6ω3) and nervonic acid (C24:1ω9), were determined by using capillary gas chromatography. High-sensitivity C-reactive protein (hsCRP) was measured by a nephelometry method. Serum level of high-molecular weight (HMW)-adiponectin was measured using a commercially available chemiluminescent enzyme immunoassay kit (Fujirebio, Tokyo, Japan). Estimated glomerular filtration rate (eGFR), an index of renal function, was calculated by an equation for Japanese [28]: eGFR(ml/min/1.73m^2^) = 194 × Cr^(‐ 1.094)^ × age^(‐ 0.287)^ × 0.739 (if female). HOMA-IR, an index of insulin resistance, was calculated by the previously reported formula: insulin (μU/ml) × glucose (mg/dl) / 405.

### Study 2: Effects of omega-3 fatty acids on expression and secretion of FABP4 in mouse 3T3-L1 adipocytes

All biochemical reagents were obtained from Sigma-Aldrich (St. Louis, MO) unless otherwise indicated. Preadipocyte 3T3-L1 cells were purchased from Health Science Research Resources Bank (Osaka, Japan). Differentiation of 3T3-L1 cells into adipocytes was induced as previously described [11]. Differentiated 3T3-L1 adipocytes were stimulated for 2 h (short-term secretion analysis) in the presence and absence of 10 μM isoproterenol or 24 h (long-term secretion and gene expression analyses) with 0–100 μM EPA or 0–100 μM DHA in Dulbecco’s Modified Eagle’s Medium (DMEM) (Invitrogen, Carlsbad, CA) supplemented with 0.5 % fatty acid-depleted BSA. The doses of reagents and incubation periods were varied according to the experimental protocol. Each experiment was performed in at least triplicate.

### Quantitative real-time PCR

Total RNA was isolated using Trizol Reagent (Invitrogen), and cDNA was synthesized using a kit (Applied Biosystems, Foster City, CA). Quantitative real-time PCR analysis using SYBR Green was performed (Applied Biosystems). The thermal cycling program was 10 min at 95°C for enzyme activation and 40 cycles of denaturation for 15 s at 95 °C, 30-s annealing at 58 °C and 35-s extension at 72 °C. Two pairs of specific primers used are as follows: 5′- AAG GTG AAG AGC ATC ATA ACC CT -3′ and 5′- TCA CGC CTT TCA TAA CAC ATT CC -3′ for FABP4, 5′- TCG CTG ATG CAC TGC CTA TG -3′ and 5′- GAG AGG TCC ACA GAG CTG ATT -3′ for peroxisome proliferator-activated receptor (PPAR)-γ2, 5′- CAA GAA CAG CAA CGA GTA CCG -3′ and 5′- GTC ACT GGT CAA CTC CAG CAC -3′ for CCAAT/enhancer binding protein α (C/EBPα) and 5′- AGT CCC TGC CCT TTG TAC ACA -3′ and 5′- CGA TCC GAG GGC CTC ACT A -3′ for 18s rRNA as an internal control gene.

### Western blot analysis

The conditioned medium (CM) isolated from adipocytes was filtered to obtain 10-50-kDa fractions of proteins using Amicon Ultra 10K and 50K devices (Millipore, Billerica, MA). For lysing cells, a cell lysis buffer, containing 50 mM Tris–HCl (pH 7.0), 5 mM EDTA, 2 mM EGTA, 10 mM Na_4_P_2_O_7_, 10 mM Na_3_VO_4_, 40 mM β-glycerophosphate, 30 mM NaF, 0.5 % NP-40, and 1 % protease inhibitor cocktail, was used. Total protein content of the cell lysate (CL) was assessed by a microplate protein assay based on the Lowry method (Bio-Rad, Hercules, CA).

Equal protein amounts per the CL and the filtered CM were subjected to SDS–polyacrylamide gel electrophoresis and electrophoretically transferred onto PVDF membranes (Whatman, Florham Park, NJ). After incubation for 1 h at room temperature with a blocking solution of 3 % BSA in Tris-buffered saline buffer containing 0.1 % Tween 20 (TBST), the membranes were incubated with primary antibodies for FABP4 (#ab13979; Abcam, Tokyo, Japan) and glyceraldehyde 3-phosphate dehydrogenase (GAPDH) (#sc-20357; Santa Cruz Biotechnology, Santa Cruz, CA) overnight at 4 °C. After washing with TBST, the membranes were incubated with a secondary antibody conjugated with horseradish peroxidase (Santa Cruz Biotechnology) for 1 h at room temperature and washed. Immunodetection was performed using a chemiluminescence kit (Roche Diagnostics, Tokyo, Japan). Densitometry analysis was performed using ImageJ software. FABP4 secretion was expressed as densitometry of FABP4 in the CM divided by those of FABP4 in the CL and GAPDH in the CL as previously described [29].

### Statistical analysis

Numeric variables are expressed as means ± SEM. For regression analyses, the distribution normality of each parameter was checked using the Shapiro-Wilk W test, and non-normally distributed parameters were logarithmically transformed. The correlation between two parameters was evaluated using Pearson’s correlation coefficient. Comparison between paired samples was done with Wilcoxon signed-rank test. For detecting significant differences in data between more than two groups, one-way analysis of variance and Tukey-Kramer *post hoc* test were used. A p value of less than 0.05 was considered statistically significant. All data were analyzed by using JMP 9 for Macintosh (SAS Institute, Cary, NC).

## Results

### Study 1

**Fig. 1:**
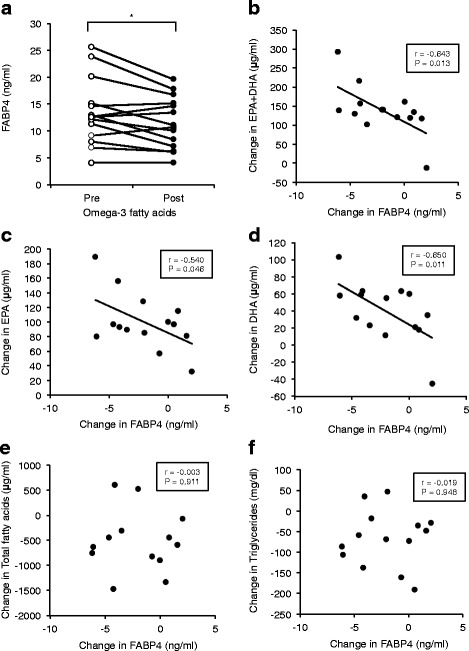
Effect of omega-3 fatty acid ethyl esters on FABP4 level (Study 1). **a**. Treatment with 4 g (2 g twice daily) omega-3 fatty acid ethyl esters, containing 1,860 mg/day eicosapentaenoic acid (EPA) ethyl ester and 1,500 mg/day docosahexaenoic acid (DHA) ethyl ester, for 4 weeks significantly decreased FABP4 levels in male patients with hypertriglycemia (*n* = 14). *P < 0.05. **b-d**. Changes (Post – Pre) in levels of EPA + DHA (**b**), EPA (**c**), DHA (**d**), total fatty acids (**e**) and triglycerides (**f**) were plotted against change in level of FABP4 for each subject

Characteristics of the patients are shown in Table 1. No one dropped out from the protocol in Study 1. Treatment with omega-3 fatty acid ethyl esters for 4 weeks significantly decreased triglycerides (163.7 ± 20.6 vs. 98.1 ± 11.4 mg/dl, *P* = 0.003), but no significant differences were found before and after treatment in waist circumference, BMI or levels of glucose, insulin, HOMA-IR, total cholesterol, LDL cholesterol, HDL cholesterol, hsCRP or HMW-adiponectin. Regarding fatty acid composition, treatment with omega-3 fatty acid ethyl esters significantly increased EPA (48.6 ± 5.3 vs. 144.6 ± 12.4 μg/ml, *P* < 0.001), DHA (139.8 ± 8.2 vs. 179.5 ± 7.6 μg/ml, *P* < 0.001), behenic acid and docosapentaenoic acid and conversely decreased palmitic acid, palmitoleic acid, oleic acid, linoleic acid, γ-linolenic acid, eicosadienoic acid, eicosatetraenoic acid, dihomo-γ-linolenic acid, arachidonic acid, docosatetraenoic acid and total fatty acids (Table 2). Serum FABP4 level was significantly decreased by 14.8 % after treatment with omega-3 fatty acid ethyl esters (13.5 ± 1.5 vs. 11.5 ± 1.1 ng/ml, *P* = 0.017) (Fig. 1a). Change (Post - Pre) in FABP4 level was negatively correlated with change in levels of EPA + DHA (r = −0.643, *P* = 0.013) (Fig. 1b), EPA (r = −0.540, *P* = 0.046) (Fig. 1c) and DHA (r = −0.650, *P* = 0.011) (Fig. 1d). However, change in FABP4 level was not correlated with change in levels of total fatty acids (r = −0.003, *P* = 0.911) (Fig. 1e), triglycerides (r = −0.019, *P* = 0.948) (Fig. 1f) or other fatty acid composition.

**Table 1:** Characteristics of the male patients (Omega-3 FAs, 4w)

	Pre	Post
n	14	
Age (years)	40.2 ± 1.7	
Body mass index (kg/m2)	25.9 ± 0.7	26.1 ± 0.7
Waist circumference (cm)	89.5 ± 1.8	89.0 ± 2.0
Biochemical data		
Total cholesterol (mg/dl)	217.2 ± 7.1	207.6 ± 8.5
HDL cholesterol (mg/dl)	57.6 ± 3.7	57.6 ± 4.3
LDL cholesterol (mg/dl)	132.6 ± 6.5	125.8 ± 8.3
Triglycerides (mg/dl)	163.7 ± 20.6	98.1 ± 11.4*
Glucose (mg/dl)	97.6 ± 2.2	98.0 ± 1.8
Insulin (μU/ml)	7.9 ± 1.9	6.5 ± 1.2
HOMA-IR	1.99 ± 0.56	1.57 ± 0.28
HbA1c (%)	5.5 ± 0.1	5.5 ± 0.1
hsCRP (mg/dl)	0.60 ± 0.17	0.75 ± 0.20
HMW-adiponectin (μg/ml)	2.42 ± 0.23	2.31 ± 0.23
FABP4 (ng/ml)	13.5 ± 1.5	11.5 ± 1.1*

Variables are expressed as n or means ± SEM

*hsCRP* high-sensitivity C-ractive protein, *HMW* high-molecular weight

*P <0.05 vs. Pre

**Table 2:** Fatty acid composition (Omega-3 FAs, 4w)

		μg/ml		%vol	
		Pre	Post	Pre	Post
Saturated fatty acids					
Lauric acid	C12:0	5.4 ± 0.9	3.4 ± 0.6	0.16 ± 0.02	0.12 ± 0.02
Myristic acid	C14:0	31.0 ± 4.2	20.7 ± 2.2	0.89 ± 0.09	0.71 ± 0.06
Palmitic acid	C16:0	607.5 ± 40.8	491.0 ± 18.3*	17.7 ± 0.4	17.0 ± 0.3
Stearic acid	C18:0	304.6 ± 20.0	265.2 ± 11.0	8.9 ± 0.3	9.2 ± 0.2
Arachidic acid	C20:0	2.0 ± 0.2	1.6 ± 0.1	0.058 ± 0.004	0.055 ± 0.004
Behenic acid	C22:0	1.1 ± 0.1	1.9 ± 0.2*	0.0077 ± 0.0077	0.092 ± 0.014*
Lignoceric acid	C24:0	0.81 ± 0.06	0.75 ± 0.03	0.025 ± 0.001	0.027 ± 0.002
Monounsaturated fatty acids					
Myristoleic acid	C14:1ω5	3.0 ± 0.6	1.4 ± 0.2*	0.083 ± 0.016	0.048 ± 0.006
Palmitoleic acid	C16:1ω7	66.2 ± 6.8	42.2 ± 3.9*	1.9 ± 0.1	1.4 ± 0.1*
Oleic acid	C18:1ω9	749.7 ± 66.9	526.7 ± 36.5*	21.6 ± 0.9	18.0 ± 0.6*
Eicosenoic acid	C20:1ω9	6.0 ± 0.7	5.3 ± 0.6	0.17 ± 0.01	0.18 ± 0.02
Erucic acid	C22:1ω9	3.4 ± 0.2	3.3 ± 0.1	0.10 ± 0.01	0.11 ± 0.01
Nervonic acid	C24:1ω9	1.9 ± 0.1	1.9 ± 0.1	0.060 ± 0.048	0.068 ± 0.005
Polyunsaturated fatty acids					
Omega-3 fatty acids					
α-Linolenic acid	C18:3ω3	26.9 ± 2.7	22.3 ± 2.8	0.78 ± 0.05	0.75 ± 0.07
Eicosapentaenoic acid (EPA)	C20:5ω3	48.6 ± 5.3	144.6 ± 12.4*	1.5 ± 0.2	5.0 ± 0.6*
Docosapentaenoic acid	C22:5ω3	19.8 ± 1.2	25.7 ± 1.4*	0.59 ± 0.04	0.90 ± 0.05*
Docosahexaenoic acid (DHA)	C22:6ω3	139.8 ± 8.2	179.5 ± 7.6*	4.2 ± 0.3	6.4 ± 0.4*
Omega-6 fatty acids					
Linoleic acid	C18:2ω6	1064.3 ± 46.2	894.0 ± 54.3*	31.6 ± 0.9	30.6 ± 1.1
γ-linolenic acid	C18:3ω6	11.0 ± 1.5	6.4 ± 1.0*	0.32 ± 0.04	0.22 ± 0.03*
Eicosadienoic acid	C20:2ω6	6.9 ± 0.5	4.7 ± 0.3*	0.20 ± 0.01	0.16 ± 0.01*
Dihomo-γ-linolenic acid	C20:3ω6	47.2 ± 3.4	27.4 ± 3.1*	1.4 ± 0.1	1.0 ± 0.1*
Arachidonic acid (AA)	C20:4ω6	248.0 ± 14.8	222.4 ± 12.1*	7.5 ± 0.4	7.8 ± 0.3
Docosatetraenoic acid	C22:4ω6	4.7 ± 0.3	2.8 ± 0.2*	0.14 ± 0.01	0.10 ± 0.01*
Omega-9 fatty acids					
Eicosatetraenoic acid	C20:3ω9	1.5 ± 0.1	1.1 ± 0.1*	0.045 ± 0.002	0.035 ± 0.002*
Total fatty acids		3402.8 ± 181.2	2899.1 ± 120.0*	100	100
Calculation					
EPA/AA		0.20 ± 0.02	0.68 ± 0.07*		
DHA/AA		0.57 ± 0.03	0.83 ± 0.04*		
EPA + DHA		188.4 ± 12.2	324.2 ± 18.6*	5.7 ± 0.5	11.4 ± 1.0*
(EPA + DHA)/AA		0.78 ± 0.05	1.50 ± 0.11*		

Variables are expressed as n or means ± SEM. *P <0.05 vs. Pre

### Study 2

**Fig. 2:**
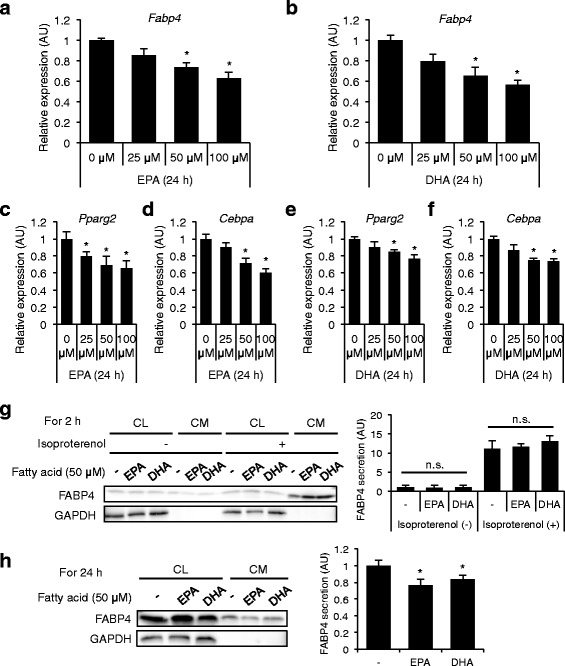
Gene expression and secretion of FABP4 in 3T3-L1 adipocytes treated with omega-3 fatty acids (Study 2). **a, b**. Gene expression of FABP4 was determined by quantitative real-time PCR in differentiated 3T3-L1 adipocytes treated with 0–100 μM eicosapentaenoic acid (EPA) (A) or 0–100 μM docosahexaenoic acid (DHA) (B) for 24 h (*n* = 3 in each group). *P < 0.05 vs. 0 μM. **c-f**. Gene expression of peroxisome proliferator-activated receptor γ2 (PPARγ2) and CCAAT/enhancer binding protein α (C/EBPα) was determined by quantitative real-time PCR in differentiated 3T3-L1 adipocytes treated with 0–100 μM EPA (c, d) or 0–100 μM DHA (E, F) for 24 h (*n* = 6 in each group). *P < 0.05 vs. 0 μM. **g**. Western blot analysis of FABP4 and glyceraldehyde 3-phosphate dehydrogenase (GAPDH) using the cell lysate (CL) and conditioned medium (CM) of 3T3-L1 adipocytes treated with 50 μM EPA or 50 μM DHA in the absence and presence of 10 μM isoproterenol for 2 h (*n* = 5 in each group). n.s., not significant. **h**. Western blot analysis of FABP4 and GAPDH using the CL and CM of 3T3-L1 adipocytes treated with 50 μM EPA or 50 μM DHA for 24 h (*n* = 5 in each group). FABP4 secretion was relatively expressed as densitometry of FABP4 in the CM divided by those of FABP4 in the CL and GAPDH in the CL. AU, arbitrary unit. *P < 0.05 vs. Fatty acid (−)

Treatment with both EPA and DHA for 24 h significantly decreased gene expression of FABP4 in 3T3-L1 adipocytes in a dose-dependent manner, and the effect tended to be more augmented with DHA treatment than with EPA treatment (Fig. 2a, b). Furthermore, both EPA and DHA significantly decreased gene expression of PPARγ2 and C/EBPα in 3T3-L1 adipocytes in a dose-dependent manner (Fig. 2c-f). Western blot analysis showed that FABP4, but not a non-secretory protein GAPDH, was present in the CM of 3T3-L1 (Fig. 2g, h), indicating that FABP4 is secreted from adipocytes without cell destruction. FABP4 secretion was induced by lipolytic stimulation with 10 μM isoproterenol, a pan-β-adrenergic agonist, for 2 h (Fig. 2g) as previously reported [11]. Treatment with 50 μM EPA or 50 μM DHA for 2 h (short-term) did not significantly change FABP4 secretion from adipocytes in the absence or presence of 10 μM isoproterenol (Fig. 2g). However, a significant decrease of FABP4 secretion from adipocytes was observed at 24 h (long-term) after treatment with 50 μM EPA or 50 μM DHA (Fig. 2h).

## Discussion

The present study demonstrated for the first time that treatment with omega-3 fatty acid ethyl esters containing EPA and DHA for 4 weeks significantly decreased serum FABP4 concentration in patients with dyslipidemia. Furthermore, *in vitro* experiments showed that treatment with EPA or DHA dose-dependently (up to 100 μM) decreased the expression and consecutive long-term secretion, but not short-term secretion, of FABP4 in 3T3-L1 adipocytes. Average changes in the levels of EPA and DHA by treatment with omega-3 fatty acid ethyl esters were 96.0 μg/ml (317 μM) and 39.7 μg/ml (121 μM), respectively, in the present study, indicating that the doses of EPA and DHA in *in vitro* experiments were physiological but not pharmacological. These findings suggest that a direct suppressive effect of omega-3 fatty acids on FABP4 expression and its consecutive secretion in adipocytes plays a role in the decrease in serum FABP4 level.

In the present study, treatment with omega-3 fatty acid ethyl esters significantly decreased triglycerides and total fatty acids and modulated levels of fatty acid composition. However, changes in the levels of triglycerides, total fatty acids and each fatty acid composition except for EPA and DHA were not correlated with reduction of FABP4 level, suggesting that qualitative, but not quantitative, change in fatty acids contributes to the reduction in FABP4 concentration. It has been reported that expression of FABP4 in adipocytes is up-regulated by PPARγ agonists and saturated and monounsaturated fatty acids [1, 2, 30–32]. On the other hand, polyunsaturated fatty acids, such as omega-3 fatty acids, have been reported to inhibit adipocyte differentiation and lipid accumulation, leading to a decrease in the expression of FABP4, also known as an adipocyte differentiation marker [25–27]. Furthermore, it has been shown that unsaturated fatty acids, including EPA, repress expression of FABP4 in RAW264.7 macrophages [33], though the predominant contributors of circulating FABP4 are adipocytes rather than macrophages [10]. In the present study, we showed that both EPA and DHA decreased FABP4 expression even in differentiated 3T3-L1 adipocytes, at least in part, via reduction in gene expression of PPARγ2 and C/EBPα, which are critical transcription factors for regulation of adipocyte differentiation [34].

There have been some reports about modulation of FABP4 concentration by drugs. Atorvastatin, a cholesterol-lowering statin, has been reported to decrease FABP4 level, but the mechanism is totally unknown [24]. It has also been shown that several angiotensin II receptor blockers (ARBs) decrease circulating FABP4 concentration [29, 35], and reduction of sympathetic nerve activation due to a class effect of ARBs, but not a direct effect of angiotensin II receptor blockade, has been postulated as a possible mechanism of decreased FABP4 level by ARBs [29], since FABP4 secretion from adipocytes is associated with β-adrenergic receptor-mediated lipolysis [10, 11]. Previous studies showed that dietary fish and omega-3 fatty acid consumption decreased sympathetic nerve activity [36, 37]. Other than direct suppressive effects of EPA and DHA on FABP4 expression in adipocytes, treatment with omega-3 fatty acid ethyl esters containing EPA and DHA may decrease FABP4 concentration by inhibiting FABP4 secretion from adipocytes associated with sympathetic tone-mediated lipolysis.

Previous studies including a recent meta-analysis have shown that both dietary and circulating EPA and DHA are associated with the low incidence of cardiovascular disease in a multiethnic population [38, 39]. Furthermore, recent clinical trials have demonstrated that omega-3 fatty acids, including EPA alone, EPA + DHA and fish oil, substantially reduce cardiovascular events [40–43]. Since there is accumulating evidence demonstrating significant roles of circulating FABP4 in insulin resistance, atherosclerosis and cardiovascular events [10, 14, 15, 17, 21–23], the results of the present study support the notion that reduction of FABP4 concentration is one of the important mechanisms by which omega-3 fatty acids prevent the development of cardiovascular disease.

The present study has several limitations. The number of patients recruited in Study 1 was small, and the possibility of a type 1 error cannot be excluded. Since it has been reported that there is a gender difference in serum FABP4 levels [9, 18], we recruited only male patients to reduce confounding factors. The present study also lacked a placebo control group. Interventional studies of placebo-control design using larger numbers of both male and female patients are necessary for evaluating the impact of omega-3 fatty acids on circulating FABP4 level. Furthermore, mouse 3T3-L1 adipocytes, a well-used cell line of adipocytes, were used *in vitro* experiments, but there might be a difference between mice and humans in regulation of the expression and secretion of FABP4 by omega-3 fatty acids. Lastly, there has been accumulating evidence indicating FABP4 is expressed in several types of cells, in addition to adipocytes and macrophages, under both physiological and pathological conditions [2, 3, 44–48]. Omega-3 fatty acids may affect the expression and secretion of FABP4 in cells other than adipocytes, though the predominant contributors of circulating FABP4 level are adipocytes rather than macrophages and other cells [3, 10].

## Conclusions

Treatment with omega-3 fatty acid ethyl esters containing EPA and DHA decreases serum FABP4 concentration in patients with dyslipidemia, at least in part, via a direct reduction in expression and consecutive secretion of FABP4 in adipocytes. Suppression of FABP4 levels may lead to the reduction of cardiovascular events as a pleiotropic effect of supplements of omega-3 fatty acids.
